# Regulatory mechanism of formaldehyde release in heme degradation catalyzed by *Staphylococcus aureus* IsdG

**DOI:** 10.1016/j.jbc.2023.104648

**Published:** 2023-03-24

**Authors:** Toshitaka Matsui

**Affiliations:** 1 Institute of Multidisciplinary Research for Advanced Materials, Tohoku University, Sendai, Japan; 2 Department of Chemistry, Graduate School of Science, Tohoku University, Sendai, Miyagi, Japan; 3 Department of Molecular and Chemical Life Sciences, Graduate School of Life Sciences, Tohoku University, Sendai, Miyagi, Japan

**Keywords:** ascorbic acid, formaldehyde, enzyme mechanism, heme oxygenase, iron metabolism, metalloenzyme, porphyrin, *Staphylococcus aureus*

## Abstract

IsdG-type enzymes catalyze the noncanonical degradation of heme to iron, staphylobilin (SB), and formaldehyde (HCHO), presumably by binding heme in an unusually distorted conformation. Their unique mechanism has been elucidated for MhuD from *Mycobacterium tuberculosis*, revealing an unusual ring opening of hydroxyheme by dioxygenation. A similar mechanism has been postulated for other IsdG enzymes; however, MhuD, which is special as an IsdG-type enzyme, retains a formyl group in the linearized tetrapyrrole. Recent reports on *Staphylococcus aureus* IsdG have suggested the formation of SB retaining a formyl group (formyl-SB), but its identification is preliminary. Furthermore, the reaction properties of formyl-SB and the mechanism of HCHO release remain unclear. In this study, the complex reaction of *S. aureus* IsdG was reexamined to elucidate its mechanism, including the identification of reaction products and their control mechanisms. Depending on the reaction conditions, IsdG produced both SB and formyl-SB as the main product, the latter of which was isolated and characterized by MS and NMR measurements. The formyl-SB product was generated upon the reaction between hydroxyheme-IsdG and O_2_ without reduction, indicating the dioxygenation mechanism as found for MhuD. Under reducing conditions, hydroxyheme-IsdG was converted also to SB and HCHO by activating another O_2_ molecule. These results provide the first overview of the complicated IsdG reaction. The heme distortion in the IsdG-type enzymes is shown to generally promote ring cleavage by dioxygenation. The presence or absence of HCHO release can be influenced by many factors, and the direct identification of *S. aureus* heme catabolites is of interest.

Bacterial heme degradation is critical for the acquisition of iron, an essential nutrient for its survival and infectivity, from host heme molecules (1, 2, 3). A family of enzymes termed heme oxygenases (HOs) is known to degrade heme into iron and biliverdin with the release of a *meso* carbon atom as carbon monoxide (CO), as shown in Fig. S1*A* (4, 5, 6, 7). The HO catalysis proceeds through three consecutive monooxygenation reactions *via* hydroxyheme and verdoheme intermediates. The substrate heme activates oxygen molecules (O_2_) for a series of unique self oxidation reactions. Until recently, the HO reaction was thought to be the only heme-degrading mechanism in biological systems. However, several novel types of heme degradation have been reported in the last decade (8, 9, 10, 11, 12). Among them, IsdG is the first enzyme family to be shown to have a noncanonical mechanism. IsdG and its paralog, IsdI, are heme-degrading enzymes in *Staphylococcus aureus*, consisting of a heme-uptake system called an iron-regulated surface determinant system (Isd system) (13). The IsdG proteins are unique in binding heme in a highly distorted conformation best described as “ruffled” (8, 14). The heme ruffling is expected to significantly modulate the O_2_ activation chemistry on the heme molecule (15). Indeed, most IsdG-type enzymes, including *S. aureus* IsdG and IsdI, degrade heme to a novel tetrapyrrole termed staphylobilin (SB), which is cleaved at the β- or δ-*meso* position and further oxidized at the diagonal *meso* carbon (β- or δ-SB shown in Fig. 1) (8, 16, 17).

**Figure 1:**
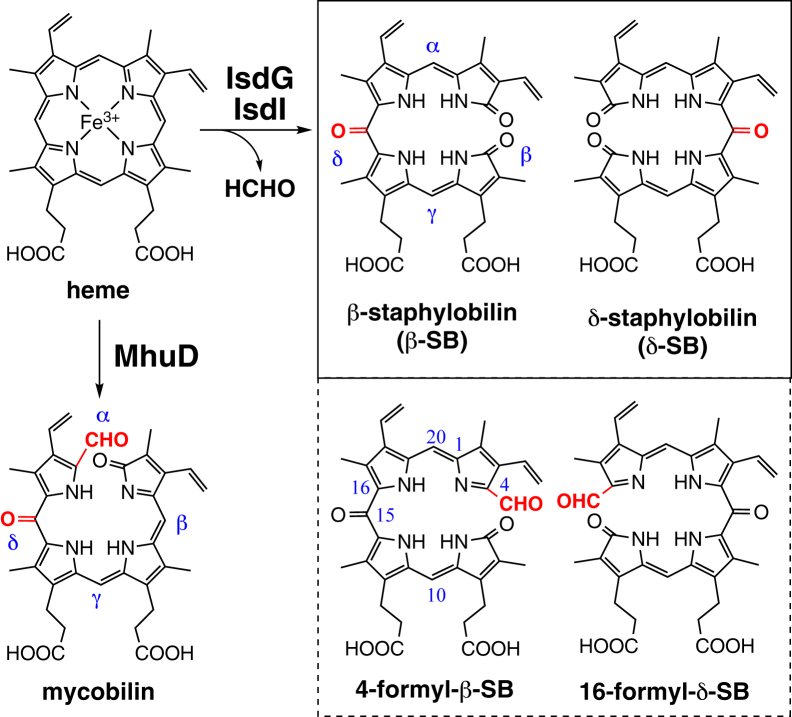
**Heme catabolites produced by IsdG, IsdI, and MhuD.** For mycobilin, only one isomer is shown for simplicity. Isd, iron-regulated surface determinant.

The unique mechanism of the IsdG family enzymes was first revealed in MhuD, an IsdG-type enzyme from *Mycobacterium tuberculosis* (18). MhuD is special in degrading heme into mycobilin having additional oxidation at the β- or δ-*meso*-carbon, similar to the SB isomers (Figs. 1 and S1*B*) (10). In contrast to SB, mycobilin is cleaved at the α-*meso* position and, more importantly, retains the carbon atom at the cleavage site as a formyl group. As expected from this retention, the MhuD reaction proceeds without the generation of CO and the verdoheme intermediate (Fig. S1) (10, 18). It has been proposed that the absence of CO formation is advantageous for *M. tuberculosis* to protect itself from host macrophages (10, 19). The first step of MhuD is shown to be the monooxygenation of the *meso* carbon atom, similar to the first step of HO (18). In the HO reaction (Fig. S1*A*), the resulting hydroxyheme is again monooxygenated at the hydroxylation site to yield verdoheme and CO. Then, the verdoheme ring is cleaved by the third monooxygenation. In contrast, MhuD cleaves the hydroxyheme ring with one O_2_ molecule through a dioxygenation mechanism in which two oxygen atoms of O_2_ are introduced into the substrate without consuming reductants (Fig. S1*B*).

The SB formation by IsdG and IsdI is coupled with the release of formaldehyde (HCHO), which indicates that their mechanism is distinct from that of canonical HO (Fig. 1) (9). Because the unusual reaction modes can be attributed to the hydroxyheme ruffling, the mechanism underlying the activity of IsdG and IsdI is thought to be similar to that underlying the activity of MhuD, despite the formation of different products. Recent mass studies have detected a new product in the IsdG reactions (20, 21). The reports claim this product to be SB retaining a formyl group at the cleavage site, termed “formyl-SB” in this manuscript (Fig. 1). The structural similarity between formyl-SB and mycobilin can support the mechanistic similarity between IsdG and MhuD. Streit *et al*. (20) also proposed that formyl-SB is stable only when complexed with IsdG and is highly destabilized with liberation from the enzyme, spontaneously releasing HCHO. Furthermore, they claimed to have detected hydroxyheme by electrospray ionization MS (ESI-MS), which was converted to the products by a rate-limiting reduction (20). However, the characterization of formyl-SB based solely on its positive mass peak at 611 is ambiguous. In addition, the structure of formyl-SB does not appear to imply the facile spontaneous release of HCHO. The properties reported for the putative hydroxyheme in IsdG differ from those observed for chemically synthesized hydroxyheme complexed with MhuD (18, 20). Thus, the current understanding of the IsdG reaction seems inadequate, and several major contradictions remain.

To this end, the complicated IsdG reaction was reexamined in this study to explore its products and their formation mechanisms. The formyl-SB isomers produced by IsdG were isolated and characterized by MS and NMR measurements. Under nonreducing conditions, hydroxyheme-IsdG was found to react immediately with O_2_ in a dioxygenation mode and exclusively produce formyl-SB. In the presence of reductants, the hydroxyheme complex was also converted to SB and HCHO by activating another O_2_ molecule. These results support the mechanistic similarity between IsdG and MhuD, likely due to the highly distorted hydroxyheme they share. The reasons for the difference in the HCHO release are discussed.

## Results

### Reaction products of IsdG

**Figure 2:**
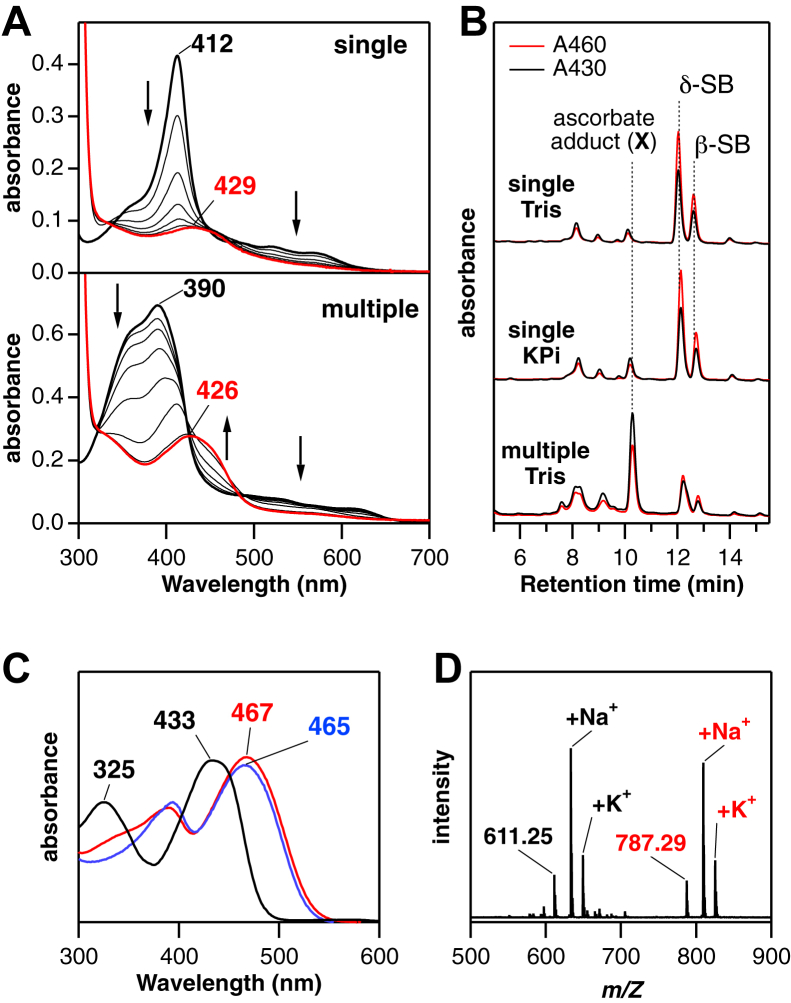
**Formation of an ascorbic acid adduct of putative formyl-staphylobilin.***A*, absorption spectral changes during single- (*top*) and multiple-turnover (*bottom*) heme degradation by IsdG in 100 mM Tris, pH 7.5 containing 5 mM ascorbic acid at 20 °C and 37 °C, respectively. *B*, HPLC chromatograms of heme catabolites under single- and multiple-turnover conditions in Tris or phosphate buffers as indicated. The products were monitored at 430 and 460 nm (*black* and *red*, respectively). *C*, online absorption spectra of the major products in the HPLC analysis. β- and δ-staphylobilin (SB) isomers (*red* and *blue*, respectively) and product X (*black*), an ascorbic acid adduct of putative formyl-SB. *D*, LC-MS spectrum of product X in a positive mode. Isd, iron-regulated surface determinant.

We first examined heme degradation by IsdG under a conventional single turnover condition where one substrate molecule in a complex with the enzyme was converted into products (8, 9). Upon addition of L-ascorbic acid as a reductant, the IsdG-heme complex in a Tris buffer, pH 7.5 at 20 °C, exhibited a significant decrease in Soret absorption (Fig. 2*A*, *top*), typical for degradation of heme. The weak absorption at 429 nm with a shoulder peak around 460 nm remained after the reaction. As previously reported (8, 9), solid-phase extraction and HPLC analysis of the reaction solution revealed the formation of two isomers of SB, β-, and δ-SB (Figs. 1 and 2*B*), with absorption peaks around 465 nm in the HPLC online spectra (Fig. 2*C*).

Next, the IsdG reaction was examined at 20 °C in 50 mM phosphate buffer, pH 7.5. Under this condition, IsdG was reported to produce a new product, which was suggested to be formyl-SB (20). In this study, although no significant difference was observed for the major products, the amounts of minor products eluting earlier than the SB isomers increased slightly (Fig. 2*B*). This observation suggests that product distribution largely depends on reaction conditions. An extensive search revealed that one of the minor products became dominant when excess heme was present, that is, under catalytic conditions (Fig. 2). Most enzymatic heme degradations have been performed under single-turnover conditions because heme-degrading enzymes are considered susceptible to product inhibition (22). However, IsdG was found to degrade at least 6 molar equivalents of heme in the Tris buffer at 37 °C (Fig. 2*A*, *bottom*). The absorption peak of free heme at 390 nm disappeared completely, and the absorption at 426 nm remained after the reaction. In the HPLC analysis, one prominent peak was observed at 10.8 min, and this major product named X showed an absorption peak at 433 nm (Fig. 2, *B* and *C*). Although the properties of product X are similar to those reported for the putative “formyl-SB” (20), further analysis reveals that X is covalently conjugated with L-ascorbic acid.

In the LC-MS analysis of X (Fig. 2*D*), a set of positive ion signals at *m/Z* 611, 633, and 649 were observed. These signals could be assigned to the proton, sodium, and potassium adducts of formyl-SB, respectively (*m/Z* calculated for [M+H]^+^ (C_34_H_35_N_4_O_7_^+^): 611.25). However, another set of three signals was observed at *m/Z* 787, 809, and 825. Relative intensities of the three signals are similar in the 611- and 787-series, and MS/MS analysis of each ion of the 787-series yielded the corresponding signal of the 611-series (Fig. S2*B*). These observations indicate that the 787- and 611-series signals originate from molecular and fragment ions of X, respectively. The mass difference between the two series, 176-Da, is equal to the molecular weight of L-ascorbic acid, suggesting its liberation upon fragmentation. The product analysis was carried out in reactions supported by two analogs of L-ascorbic acid to confirm the presence of L-ascorbic acid in X (Fig. S2*A*). In both reactions, one prominent peak was detected at varied retention times (Fig. S2*C*). The fragmented 611-series mass signals were observed for all the products, and the loss in mass upon the fragmentation was equal to the molecular weights of the two analogs used (Fig. S2, *A* and *D*). These findings indicate that product X is the ascorbic acid adduct, most likely the ascorbic acid adduct of formyl-SB.

### Formation and characterization of formyl-SB

**Figure 3:**
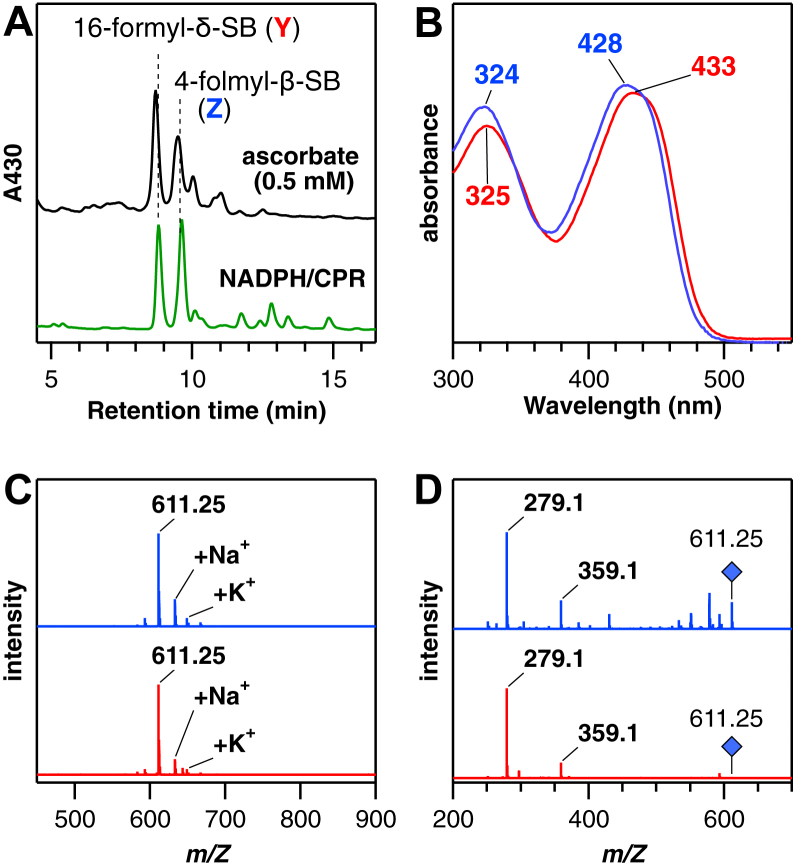
**Formation of formyl-staphylobilins.***A*, HPLC chromatograms of IsdG products obtained with a low concentration of L-ascorbic acid (*black*, 0.5 mM) or NADPH/cytochrome P450 reductase (CPR) (*green*, 125 μM/0.1 μM, respectively) in 0.1 M potassium phosphate, pH 7.0 at 37 °C. *B*, online absorption spectra in the HPLC analysis, (*C*) LC-MS spectra, and (*D*) MS/MS spectra of two new products, Y and Z (*red* and *blue*, respectively). All MS and MS/MS spectra were measured in a positive mode, and the 611.25 species (*blue rhombus*) were subjected to the MS/MS analysis. Isd, iron-regulated surface determinant.

The conjugation of ascorbic acid is likely an artifact of using a high concentration of ascorbic acid in heme degradation. At lower ascorbic acid concentrations (0.5 mM), the catalytic IsdG reaction resulted in two new major products eluting at 8.8 min (Y) and 9.6 min (Z) (Fig. 3*A*, *top*). The same products were also produced using NADPH and cytochrome P450 reductase (CPR), a cognate reductase for mammalian HO, as a reducing system (Fig. 3*A*, *bottom*). The two new products with absorption peaks at around 430 nm exhibited the 611-series but not the 787-series mass signals (Fig. 3, *B* and *C*), implying that these are formyl-SBs without modification. Retention of the carbon atom at the cleaving *meso*-position is suggested by the low yield of formaldehyde (9.7 ± 2.5 % of hemin degraded) in the CPR-dependent degradation. In the ^18^O_2_-labeling experiment, the putative formyl-SB showed a mass increase of 4 and 6 Da (Fig. S3). The increase is consistent with the formyl-SB structure, where three oxygen atoms are incorporated upon the heme degradation, one of which is a water-exchangeable formyl oxygen atom (Fig. 1). There are eight isomers for formyl-SB, grouped into three by fragmentation patterns (Fig. S4). Major MS/MS fragments of Y and Z were detected at *m/Z* 279 and 359 (Fig. 3*D*); these products are suggested to be either 4-formyl-β-SB or 16-formyl-δ-SB (Fig. S4).

**Figure 4:**
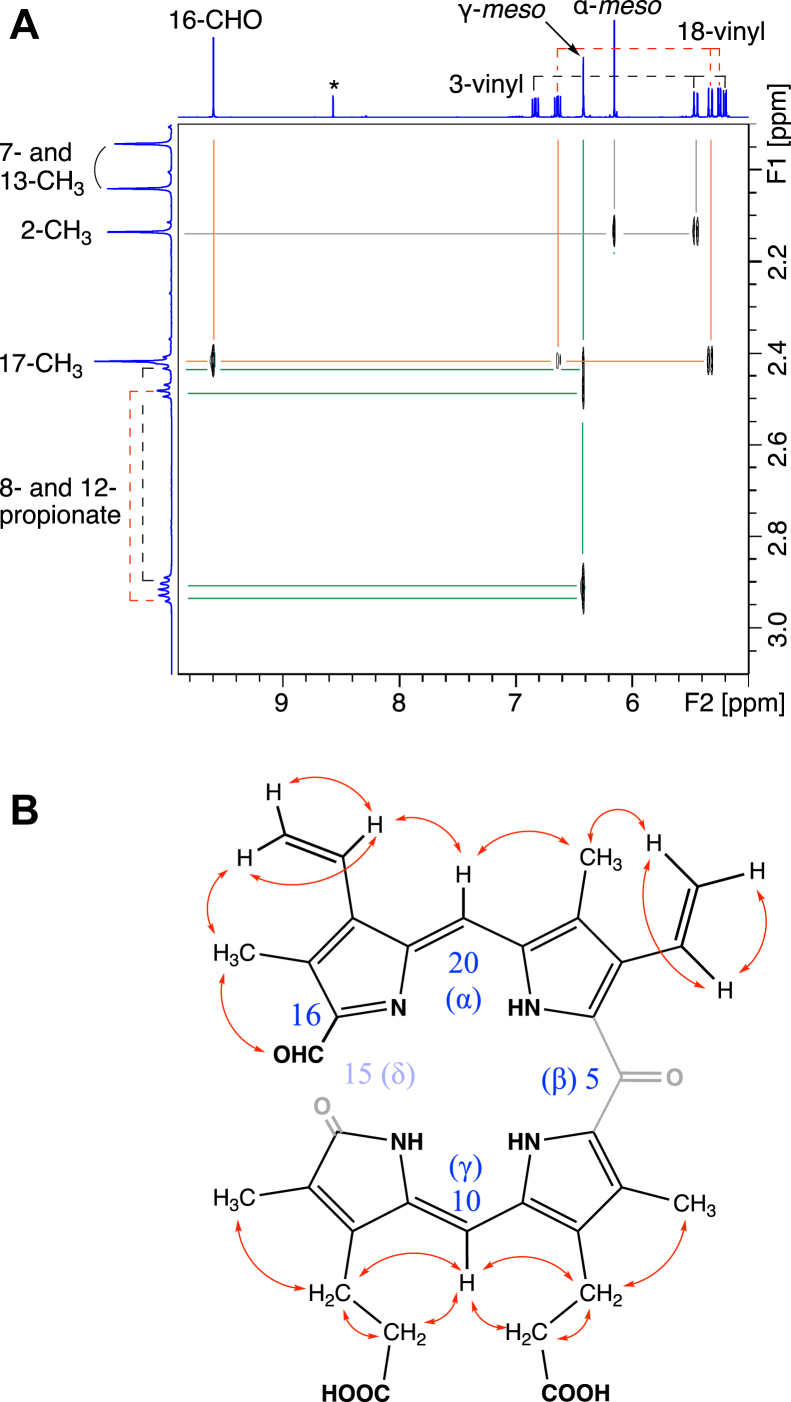
**Characterization of 16-formyl-δ-SB (product Y).***A*, NOESY spectrum (selected region) and (*B*) a molecular structure with observed NOE correlations. SB, staphylobilin.

The products Y and Z were prepared *via* large-scale enzymatic reactions and purified using HPLC for structural determination by NMR. As shown in Figs. 4 and S5, product Y showed a proton signal of the formyl group at 9.60 ppm (^13^C: 180 ppm in hetero-nuclear single quantum coherence). This formyl proton exhibited NOE correlation with methyl protons, which are then connected with vinyl protons (Fig. 4*A*). The vinyl protons showed sequential correlation with *meso*, methyl, and another vinyl group protons (Figs. 4 and S5). The observation is consistent with the upper-half structure of 16-formyl-δ-SB. The lower-half structure is consistent with the observed NOE correlations of methyl, methylene, *meso*, methylene, and methyl protons (Fig. S5, *C* and *D*), even though 7- and 13-methyl protons and 8- and 12-methylene protons were not distinguishable due to its symmetric structure. Based on these findings, product Y is identified as 16-formyl-δ-SB. Similarly, product Z was identified as 4-formyl-β-SB based on NOE correlations leading from a formyl proton to vinyl and then to methyl protons (Fig. S6). These results unequivocally characterize 4-formyl-β-SB and 16-formyl-δ-SB as the major products of IsdG.

### Formation mechanism of formyl-SB

**Figure 5:**
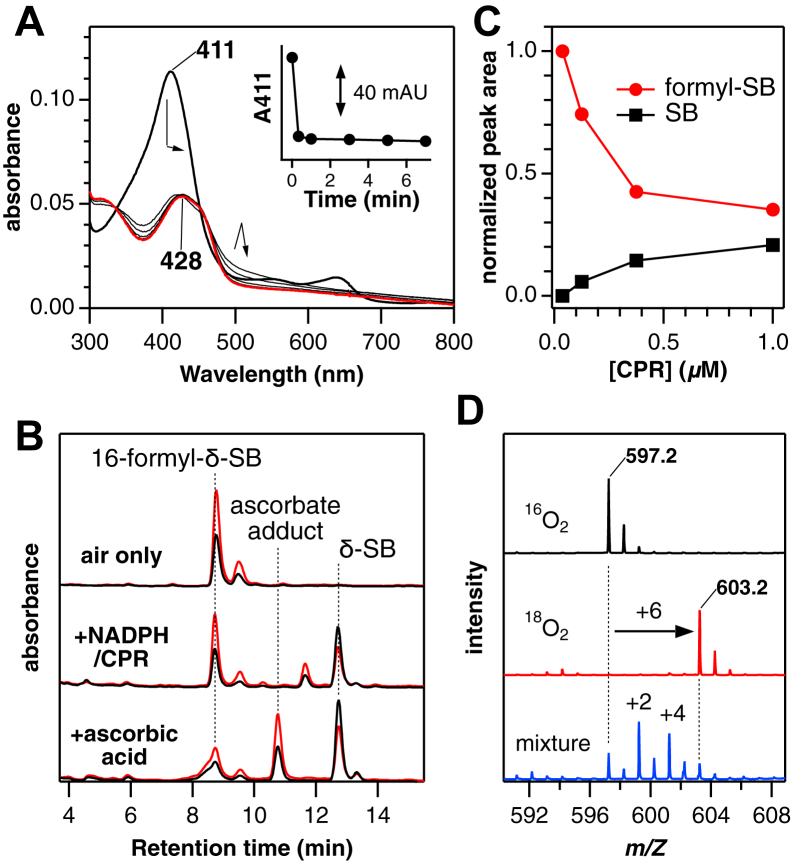
**Staphylobilin formation induced by reduction.***A*, absorption spectral change in the reaction of β-hydroxyheme-IsdG with O_2_ at 30 °C in 0.1 M potassium phosphate, pH 7.5. (*inset*) Absorbance changes at 411 nm. *B*, HPLC chromatograms of air-oxidized β-hydroxyheme-IsdG monitored at 465 and 430 nm (*black* and *red*, respectively). 0.5 μM CPR or 5 mM ascorbic acid was added for the reducing condition. *C*, product change in the multiple-turnover aerobic heme degradation at 37 °C with varied concentrations of CPR. The total peak areas of SB and formyl-SB isomers were normalized to that of formyl-SB at the lowest CPR concentration. *D*, ESI-MS spectral change of SB upon isotope labeling. SB, staphylobilin; Isd, iron-regulated surface determinant; ESI-MS, electrospray ionization MS; CPR, cytochrome P450 reductase.

**Figure 6:**
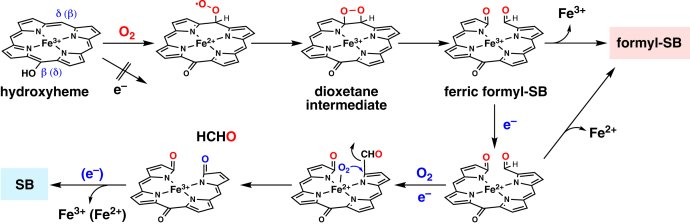
**Proposed formation mechanism of SB and formyl-SB by IsdG.** Isd, iron-regulated surface determinant; SB, staphylobilin.

Formyl-SBs are regioisomers of mycobilin (Fig. 1), and their formation mechanism should be similar to that of MhuD (10, 18). MhuD has been shown to initially activate O_2_ with two electrons and mono-oxygenate the heme *meso*-carbon. The resulting hydroxyheme intermediate is cleaved at the nonhydroxylated *meso*-carbon *via* a dioxygenation mechanism, that is, one O_2_ molecule inserts two oxygen atoms without consuming any reductant. The β-*meso*-hydroxyheme– and δ-*meso*-hydroxyheme–IsdG complexes were prepared anaerobically, and their reactions with O_2_ were studied to determine the mechanism. As in MhuD (18), β-hydroxyheme in IsdG reacted immediately with O_2_ (within 20 s), causing a significant decrease in Soret absorbance probably by breaking the porphyrin π-conjugation system (Fig. 5*A*). The rapid initial phase is followed by a small spectral change in a visible region over ca. 5 minutes. HPLC analysis of the reaction mixture reveals 16-formyl-δ-SB as a major product (Fig. 5*B*). Similarly, the reaction of δ-hydroxyheme-IsdG with O_2_ in the absence of reductant produced 4-formyl-β-SB (Fig. S7, *A* and *B*). Neither δ-SB nor β-SB was observed in these nonreducing reactions. These findings show that β-hydroxyheme– and δ-hydroxyheme–IsdG complexes react with O_2_ to form the formyl-SB isomers. The air oxidation occurs at the *meso* position, diagonal to the hydroxylated site, to cleave the porphyrin ring (Fig. 6). Furthermore, the absence of a reductant requirement for formyl-SB production supports ring opening by the dioxygenation mechanism, as reported for MhuD.

### HCHO release induced by reduction

The products of the hydroxyheme reaction changed under reducing conditions (Fig. 5*B*). Air-oxidation of β-hydroxyheme in the presence of NADPH and CPR produced 16-formyl-δ-SB and δ-SB. When ascorbic acid was added as the reductant, the ascorbic acid adduct of putative formyl-SB was observed in addition to the above two products. Thus, hydroxyheme is the common intermediate for all three types of products observed in the IsdG catalysis (Figs. 2 and 3). Similarly, in the catalytic heme degradation, increasing the CPR concentration produced more SB and less formyl-SB (Fig. 5*C*), indicating that the reduction promoted the release of HCHO. The product-determining reduction would take place after the reaction of hydroxyheme with O_2_, because under anaerobic conditions, the ferric β-hydroxyheme–IsdG complex was hardly reduced by NADPH/CPR or ascorbic acid (Fig. S7*C*).

^18^O_2_ labeling experiments revealed that all three oxygen atoms inserted into SB originated from O_2_ and did not exchange with the water oxygen (Fig. 5*D*). The two possible modes of the oxygen insertion are (Fig. S8*D*): one atom from each of *three* O_2_ molecules (“O × 3” mode) or one and two atoms from *two* O_2_ molecules regardless of order (“O+2O” mode). In order to discriminate the two reaction modes, the SB labeling was also performed with a mixture of ^16^O_2_ and ^18^O_2_. When the amounts of ^16^O_2_ and ^18^O_2_ were similar, the mass increased by 2 and 4 Da, with reduced increases of 0 and 6 Da (Figs. 5*D* and S8*A*). The distribution is rationalized by the “O×3” mode and not by the “O+2O” mode. Moreover, in the two experiments with different ^16^O_2_/^18^O_2_ ratios, the observed mass intensity of SB can be reproduced only by the “O×3” mode (Fig. S8, *B* and *C*). Therefore, the SB formation should proceed through, at least, three O_2_ reactions. The first and second O_2_ molecules would be used for the formation and reaction of *meso*-hydroxyheme. It is likely that the third O_2_ molecule is reductively activated and inserts one oxygen atom at the ring cleavage site while releasing HCHO (Fig. 6).

## Discussion

### Characterization of formyl-SB

In this study, the complicated reactions of *S. aureus* IsdG were elucidated through product identification and reaction analysis of the key hydroxyheme intermediate. It has been shown that IsdG and IsdI degrade heme into SB and HCHO (8, 9). This study reveals that IsdG can produce SB and formyl-SB as the main products, depending on the reaction conditions (Figure 1, Figure 3, Figure 4 and S3–S6). Although it was isolated and structurally determined for the first time in this study, Streit *et al*. suggested the formyl-SB formation using a direct ESI-MS analysis of the IsdG reaction solution (20). They proposed that unstable formyl-SB is not the final product but spontaneously releases HCHO to form SB when liberated from the IsdG protein. However, formyl-SB in this study was stable for isolation and characterization, as expected from its chemical structure (Fig. 1) and as found for mycobilin produced by MhuD (10). No significant decomposition was observed during the large-scale preparation process for NMR measurement for approximately 1 week. Even in the catalytic heme degradation, where the protein-free products appear to be exposed to various chemicals, including reactive oxygen species, formyl-SB was observed as the major product (Fig. 3*A*), with only small amounts of HCHO released.

Although the cause of the large discrepancy in the product stabilities is unknown, careful product identification is required. The unexpected formation of the ascorbic acid adduct of putative formyl-SB was observed in the IsdG reaction using a high concentration of ascorbic acid (Figs. 2 and S2). Since the adduct readily fragments to release ascorbic acid and shows the same MS signal as formyl-SB at *m/Z* 611, its unambiguous detection requires lower ionization energies and the removal of impurity-derived signals by LC-MS (Fig. 2*D*). Although direct MS analysis of enzyme reaction solutions may provide new insights (20), observations can be negatively influenced by interference from proteins and various reaction components.

### Formation mechanism of formyl-SB

As expected from the structural similarity with mycobilin (Fig. 1), formyl-SB is produced by IsdG through a mechanism nearly identical to that of mycobilin formation by MhuD (18). The reaction analysis using chemically synthesized β- and δ-hydroxyheme revealed that the hydroxyheme complexed with IsdG reacts rapidly with O_2_ at nonhydroxylated *meso*-position and is cleaved by dioxygenation without consuming reducing equivalents (Figs. 5, 6, and S7). The ring opening in IsdG is likely to proceed through a dioxetane intermediate as proposed for MhuD (Figs. S1*B* and 6). Recent quantum calculations of the MhuD reaction have supported the above-proposed mechanism (23). The special reactivities of hydroxyheme common to MhuD and IsdG would be a general effect of its severe distortion, leading to unique CO-free heme degradation.

Streit *et al*. reported the ESI-MS detection of ferric hydroxyheme in IsdG and its conversion to the product by slow reduction (20). However, it is unlikely that hydroxyheme can be detected by MS measurements in atmospheric conditions considering its high reactivity with O_2_ (Figs. 5*A* and S7*A*). In addition, because ferric hydroxyheme was not reduced by either ascorbate or NADPH/CPR before reacting with O_2_ (Fig. S7*C*), the reducible species observed should have a different mass than hydroxyheme. Therefore, the species detected in the previous MS study is unlikely to be hydroxyheme. This observation emphasizes the importance of direct reaction analysis using synthetic intermediates.

Formyl-SB formation appears to be enhanced in catalytic reactions compared to single turnover experiments (Figs. 2 and 3). The obvious difference in catalytic conditions is the presence of excess heme, which may interact with the Fe–formyl-SB complex of IsdG to facilitate its release and subsequent Fe release. This proposal is supported by a report that *M. tuberculosis* MhuD can bind up to two molecules of heme to its active site (24). However, only single heme binding has been reported for IsdG. Alternatively, the released Fe iron may bind to IsdG and modulate its reaction. Further investigation is required to explore the mechanism for the preferential formation of formyl-SB.

One of the remaining questions about the IsdG/MhuD reaction is the mechanism by which O_2_ reacts with the nonhydroxylated *meso*-carbon of hydroxyheme. The MhuD products, two isomers of mycobilin, are cleaved at the α-*meso* position (Fig. S9*A*). The two isomers are thought to be generated because the heme is inverted along its pseudo-twice symmetric axis (α-γ *meso*-positions) and binds to the protein in the two inverted conformations. In both binding modes, α-*meso*-carbon is expected to be at a similar position in the protein (*blue arrow* in Fig. S9*A*). This indicates that dioxygenation occurs exclusively in this vicinity, suggesting the importance of peripheral structure in controlling regioselectivity. In contrast, the heme is bound to the IsdG protein with a 90-degree rotation around the heme normal compared to MhuD (Fig. S9*B*). Thus, the dioxygenation at the region corresponding to the putative dioxygenation site of MhuD can explain the β- or δ-*meso* cleavage of formyl-SB (*blue arrow* in Fig. S9*B*). However, even reverse selectivity can explain the β-/δ-oxidations in formyl-SB. To further understand the unique heme degradation by the IsdG-type enzymes, it is important to clarify the role of the proteins in regulating the regioselectivity of the second dioxygenation as well as the first *meso*-hydroxylation.

### Formation mechanism of SB and HCHO

The formation of SB and HCHO appears to require one more reductive O_2_ activation than the formyl-SB formation. The SB formation under catalytic conditions became more pronounced with faster reduction (Fig. 5*C*), and the three inserted oxygen atoms were found to originate from three O_2_ molecules (Figs. 5*D* and S8). It is obvious that the first O_2_ molecule is used to form *meso*-hydroxyheme. The resulting ferric hydroxyheme is not easily reduced (Fig. S7*C*) and should react with the second O_2_ molecule. Since the reaction up to this point is common to the formyl-SB formation, the second O_2_ should react at the *meso*-position diagonal to the hydroxylated site, probably in the dioxygenation mode. During the dioxygenation and prior to iron liberation, the critical reduction should occur to release HCHO *via* the third O_2_ activation. A good candidate for the intermediate undergoing reduction is the ferric formyl-SB complex (Fig. 6). If the reduced ferrous complex binds O_2_ and activates it by further reduction, the terminal oxygen may attack the α-pyrrole carbon, inserting an oxygen atom instead of expelling the CHO group to form SB. This mechanism is in good agreement with the oxygen source of SB (Figs. 5*D* and S8). Unfortunately, free HCHO can exchange oxygen quickly with water molecules, making it difficult to ascertain the dioxygenation mechanism in the SB formation.

If the proposed mechanism is correct, the CPR concentration dependence on SB formation should be caused by the competition between ferric formyl-SB reduction and ferric iron’s liberation (Figs. 5*C* and 6). In addition, the reason why SB does not reach 100% even at CPR saturation may be a result of competition between the O_2_ binding to and the release of ferrous iron (Figs. 5*C* and 6). In the case of MhuD, the only reported product is mycobilin which retains a formyl group (18). The absence of HCHO release can be attributed to a very slow reduction of the putative ferric–mycobilin complex, whose conversion to mycobilin requires the addition of a potent chelator for ferric iron (deferoxamine) (18). The distinct redox properties of the iron-product complexes in MhuD and IsdG may be attributed to the difference in the chemical structure and/or distortion of the linearized tetrapyrroles (Fig. 1). These arguments, however, do not rule out HCHO release by MhuD. Figure 6 suggests that if the reduction is greatly accelerated, such as by increasing the reductase concentration or using other reductases, the HCHO release (or a corresponding reaction) should occur even in MhuD. It is important to analyze the MhuD reaction under a wider range of conditions to investigate whether there are fundamental functional differences from IsdG.

### Actual heme catabolites in *S. aureus* and *M. tuberculosis*

The *in vitro* reaction analysis in this study showed that the major products of IsdG can be either SB or formyl-SB, depending on the reaction conditions. This finding raises the question of which is the actual heme catabolite of *S. aureus.* Although the main purpose of heme degradation in pathogenic bacteria is thought to be the acquisition of iron (1, 2, 3), the IsdG/IsdI reaction has also been noted as a source of formaldehyde in *S. aureus* (24).

SB and HCHO formation was pronounced at lower temperatures under single-turnover conditions, whereas formyl-SB was abundant at 37 °C under multiple-turnover conditions (Figs. 2 and 3). The latter condition appears closer to physiological conditions, suggesting that the actual product of IsdG is formyl-SB. The advantage of this pathway is that toxic HCHO is not produced. However, one important determinant of the IsdG product is the reduction rate (Fig. 5*C*). The cognate reductases of IsdG and IsdI are proposed to be IruO and NtrA (25, 26), and reduction in *S. aureus* may be much faster to predominate SB formation. The highly reactive HCHO may have some physiological role, although there are no clear reports for *S. aureus* at this time. Tetrapyrrole products in mammals and plants are used as antioxidants and protein pigments (27, 28). The release of HCHO may modulate the redox properties of SBs and their conjugation to proteins, while the biological functions of the tetrapyrrole products have not yet been identified in bacteria. To further discuss the biological significance of the unique heme degradation, the direct detection and determination of the heme catabolites produced by *S. aureus* are required. As discussed above, this study also suggests the presence of an alternative reaction of MhuD, especially *in vivo* where a cognate reductase is present, indicating the importance of determining the *in vivo* product of *M. tuberculosis* as well. If the *in vivo* reaction modes of IsdG and MhuD are different, it is of great interest to elucidate how these closely related enzymes differentially regulate heme degradation.

## Experimental procedures

### Materials

The preparation of IsdG, its heme complex, and human CPR was performed as previously reported (9, 29). Hydroxyheme was synthesized and reconstituted with IsdG in an anaerobic glove box (MBraun, UNIlab) as reported for MhuD (18, 30, 31). Catalase and hemin chloride were purchased from Sigma-Aldrich. ^18^O_2_ was purchased from Spectra Stable Isotope (^18^O content: >95%). Other chemicals obtained from Wako Chemicals, Nakalai Tesque, and Oriental Yeast were used without further purification.

### Heme degradation and tetrapyrrole analysis

Heme degradation by IsdG was performed mainly under multiple-turnover conditions at 37 °C in the presence of 2 μM heme–IsdG complex, 10 μM hemin, and 1 μM catalase. The reaction mixture contained L-ascorbic acid, its analogs, or NADPH/CPR as reducing agents at the indicated concentrations. In the single-turnover reactions, 4 μM of the heme–IsdG complex was incubated with 5 mM L-ascorbic acid and 1 μM catalase. The absorption spectral change during the reaction was observed by an Agilent 8453 spectrophotometer. All the reactions for ^18^O_2_-labeling studies and hydroxyheme-IsdG were carried out in the anaerobic glove box at 30 °C, with monitoring absorption spectra using a Shimadzu UV1500 spectrophotometer. O_2_ was injected with a gas-tight syringe or added as air-saturated water. Solid phase extraction of colored tetrapyrroles was performed using LC-8 SPE columns (0.1 g, Supelco) as previously reported (10). The effluents were analyzed on a Shimadzu LC-20 HPLC system equipped with a Tosoh ODS-100V reversed-phase column (3 × 150 mm) over 15 min at a flow rate of 0.5 ml/min using a linear gradient from 55% methanol/45% 0.1 M ammonium acetate (v/v) to 75% methanol (v/v). ESI-MS and LC-MS analyses were performed on a micrOTOF-Q II mass spectrometer (Bruker Daltonics) equipped with an Agilent 1260 HPLC system (10). For HCHO quantitation, multiple-turnover heme degradation supported by NADPH/CPR was performed with 20 μM hemin and 4 μM heme-IsdG. Derivatization and quantitation of HCHO by an acetylacetone method were performed as described elsewhere (9, 32).

### Large-scale preparation of formyl-SB

The large-scale synthesis of formyl-SB isomers was carried out at 37 °C in 20 mM Bicine, pH 8.0. The reaction mixture contained 5 μM heme-IsdG, 50 μM hemin, and 1 mM L-ascorbic acid. After 100 min, one-third volume of saturated guanidine hydrochloride was added to the solution. The yellow pigments were collected by repetitive solid-phase extraction on an LC-18 SPE column (0.5 g, Supelco). The crude products were purified using the LC-20 HPLC system equipped with an FRC-10A fraction collector. Separation was performed on a Tosoh ODS-100V reversed-phase column (4.6 × 150 mm) over 30 min at a flow rate of 1 ml/min using a linear gradient from 48% methanol/52% 0.1 M ammonium acetate (v/v) to 52% methanol (v/v). Each isolated product was dried and redissolved in deuterated methanol (methanol-*d*_4_). The NMR spectra of the purified formyl-SB isomers were measured using a Bruker Avance III 600 spectrometer with a CryoProbe ATM.

## Data availability

Data generated in this study are included in the article or supporting information.

## Supporting information

This article contains supporting information.

## Conflict of interest

The author declares that he has no conflicts of interest with the contents of this article.
