# Supplementary material for: Regulatory mechanism of formaldehyde release in heme degradation catalyzed by Staphylococcus aureus IsdG
Source: J Biol Chem. 2023 Mar 24;299(5):104648. doi: 10.1016/j.jbc.2023.104648 (PMC10148152; doi:10.1016/j.jbc.2023.104648)
Supplement: Supplemental data [file mmc1.pdf]

Supporting Information

Regulatory mechanism of formaldehyde release in heme degradation catalyzed by Staphylococcus aureus IsdG

Toshitaka Matsui[1,2,3*]

From [1]Institute of Multidisciplinary Research for Advanced Materials, Tohoku University, 2-1-1 Katahira, Aoba, Sendai 980-8577, Japan; [2]Department of Chemistry, Graduate School of Science, Tohoku University, 6-3 Aramaki Aza-Aoba, Aoba, Sendai, Miyagi 980-8578, Japan; [3]Department of Molecular and Chemical Life Sciences, Graduate School of Life Sciences, Tohoku University, 2-1-1 Katahira, Aoba, Sendai, Miyagi 980-8577, Japan

The supporting information contains

Figures S1–S9

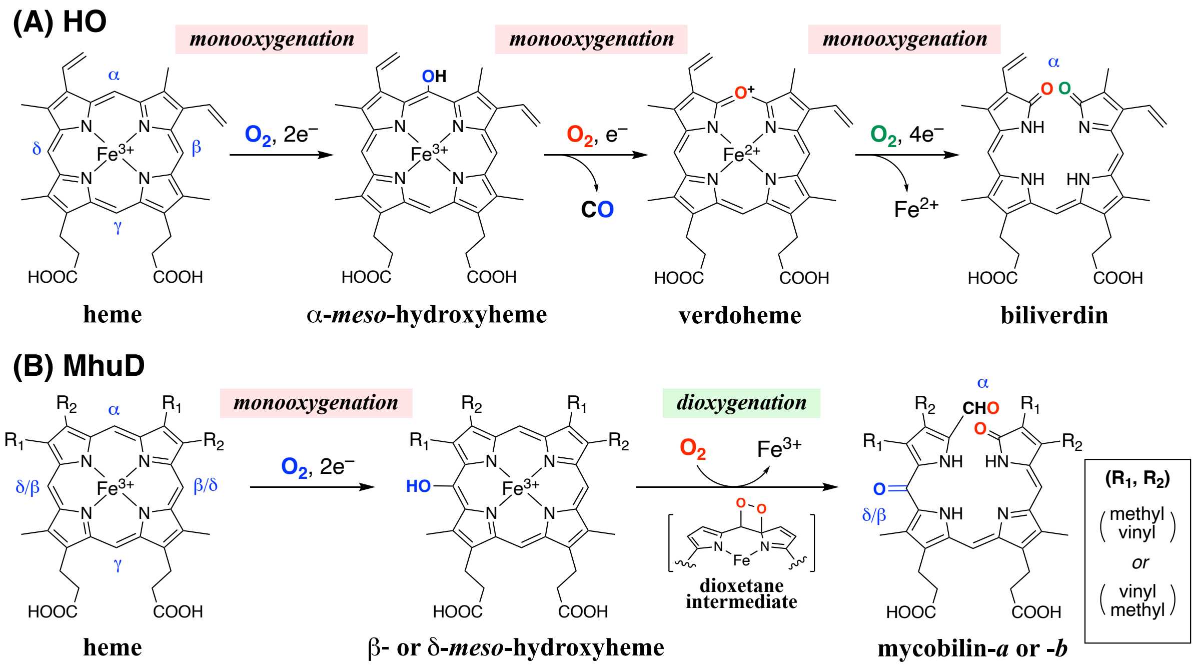

Figure S1. Heme degradation mechanisms of heme oxygenase (HO) (A) and MhuD (B).

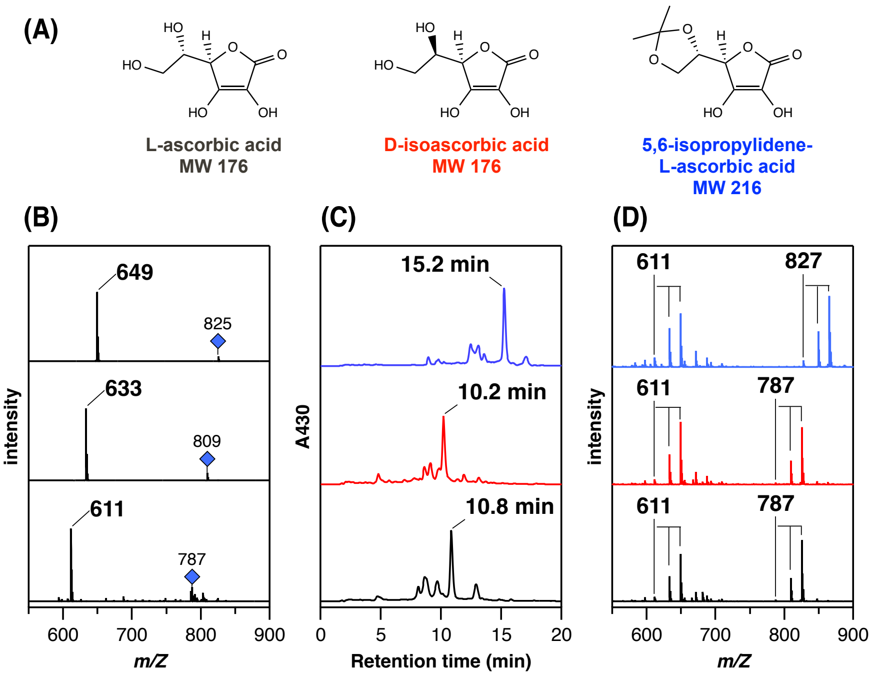

Figure S2. Heme catabolites of IsdG under the multiple-turnover condition with high concentrations (2 mM) of ascorbic acid and its analogs. (A) Structures and molecular weights of L-ascorbic acid and its analogs used. (B) MS/MS spectra of the product X obtained with L-ascorbic acid. The selected ion for each MS/MS analysis is indicated by a *blue rhombus*. (C) HPLC chromatograms and (D) ESI-MS spectra at the major peaks of the IsdG products obtained with L-ascorbic acid (*black*), D-isoascorbic acid (*red*), and 5,6-isopropylidene-L-ascorbic acid (*blue*). Three signals connected with lines in panel (D) indicate a set of H^+, Na^+, and K^+ adducts of the same species. Mass values are indicated only for the proton adducts. All samples for (B)–(D) were prepared by reactions in 0.1 M potassium phosphate, pH 7.0, at 37 °C.

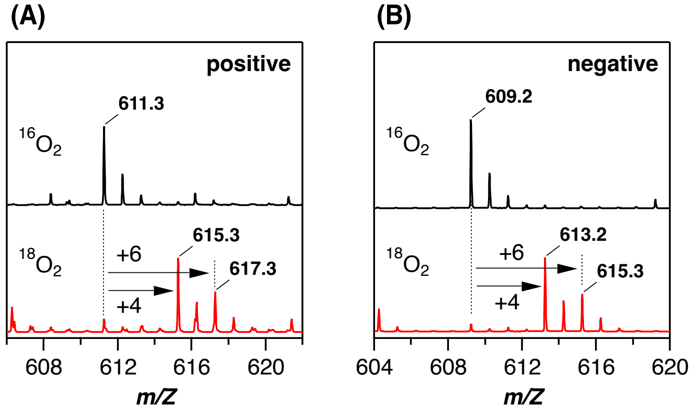

Figure S3. ESI-MS spectral changes of formyl staphylobilin (formyl-SB) upon $^{18}O_2$-labelling. (A) positive- and (B) negative-ion modes. The samples were prepared via the single-turnover degradation of 6 μM heme-IsdG with 1.25 mM ascorbic acid in the anaerobic glove box.

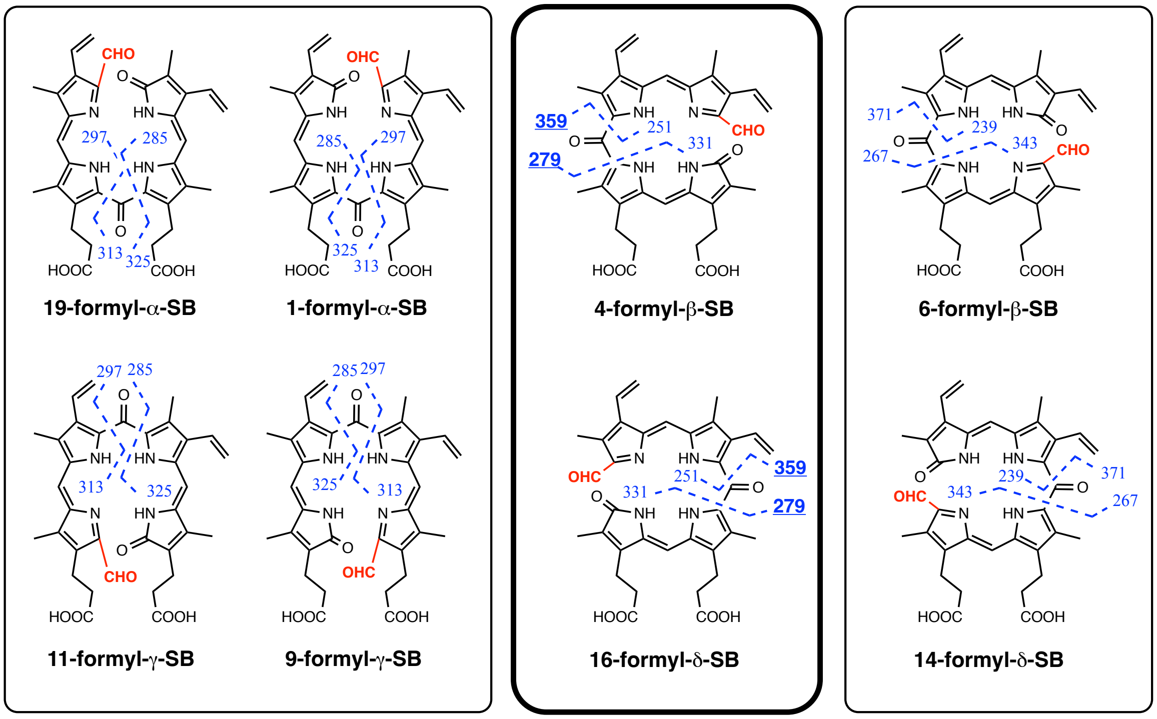

Figure S4. Eight isomers of formyl staphylobilins (formyl-SB) and their possible fragmentation patterns.

(A)

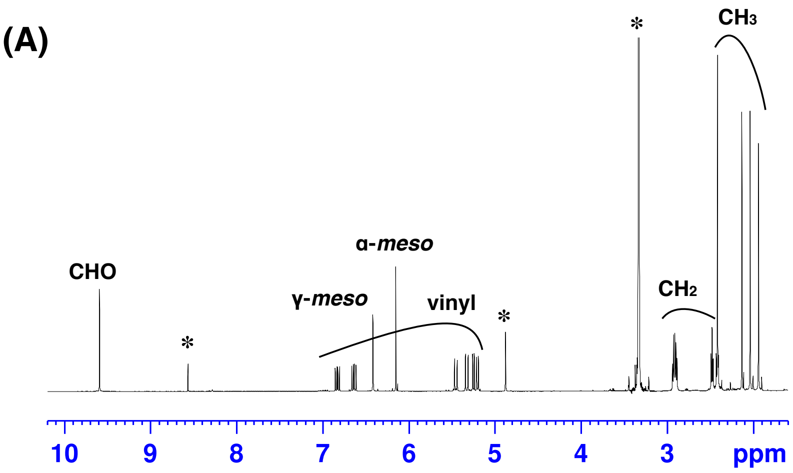

^{1}H-NMR (600 MHz, CD_3OD): δ = 9.60 (s, 1H, 16-CHO), 6.84 (dd, J = 18.1, 11.6 Hz, 1H, 3-vinyl-CH), 6.65 (dd, J = 17.9, 11.6 Hz, 1H, 18-vinyl-CH), 6.43 (s, 1H, 10C-H), 6.16 (s, 1H, 20C-H), 5.46 (dd, J = 18.1 and 1.8 Hz, 1H, 3-vinyl-CH_2, *trans*), 5.34 (dd, J = 17.9 and 1.6 Hz, 1H, 18-vinyl-CH_2, *trans*), 5.26 (dd, J = 11.6 and 1.6 Hz, 1H, 18-vinyl-CH_2, *cis*), 5.21 (dd, J = 11.7 and 1.7 Hz, 1H, 3-vinyl-CH_2, *cis*), 2.93 and 2.49 (m, 4H, 8- or 12-CH_2), 2.91 and 2.43 (m, 4H, 12- or 8-CH_2), 2.42 (s, 3H, 17-CH_3), 2.14 (s, 3H, 2-CH_3), 2.05 and 1.95 (s, 3H, 7- and 13-CH_3)

(B)

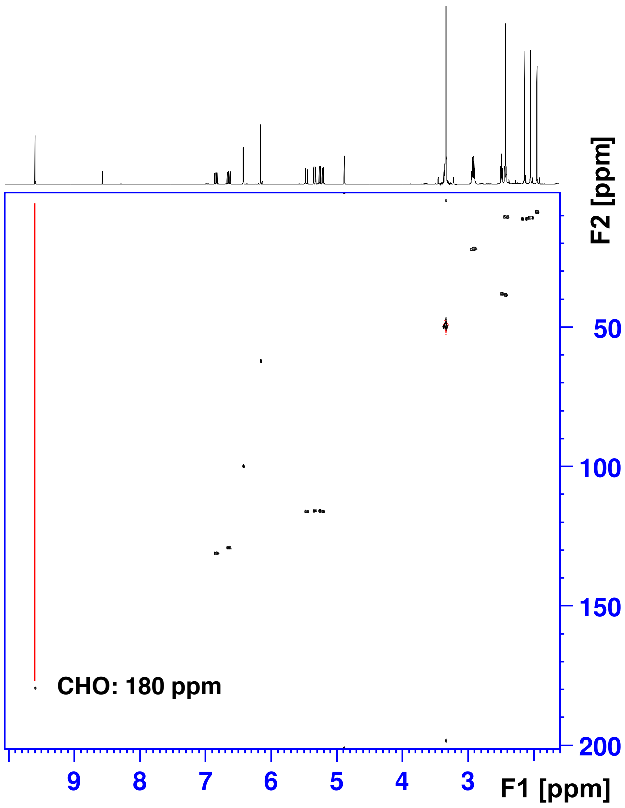

Figure S5. NMR spectra of 16-formyl-δ-staphylobilin (product Y). (A) ^{1}H-NMR and (B) HSQC spectra (*continued*).

(C)

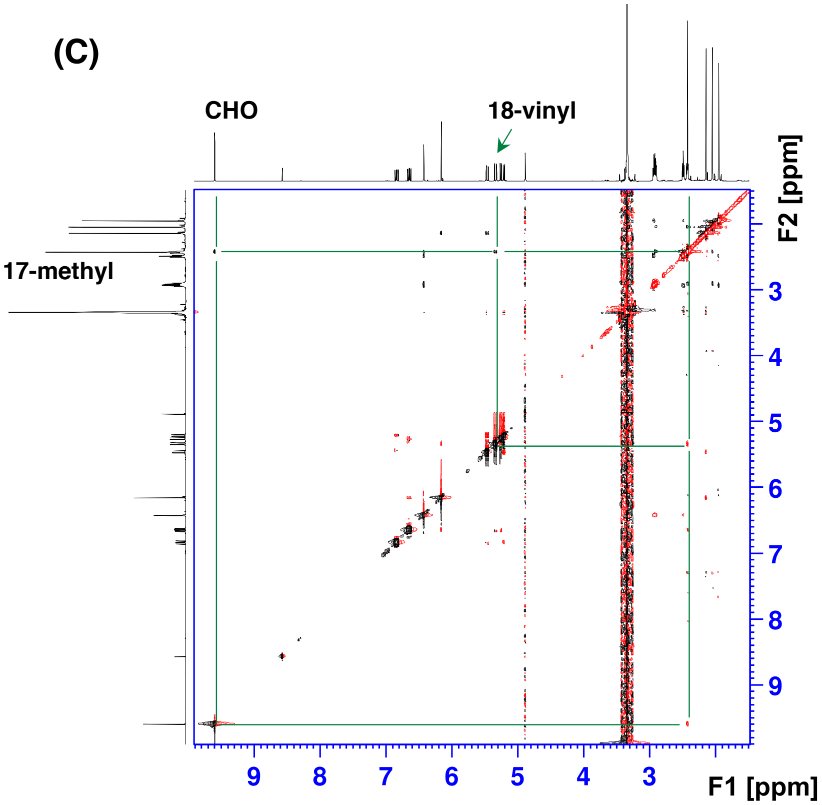

(D)

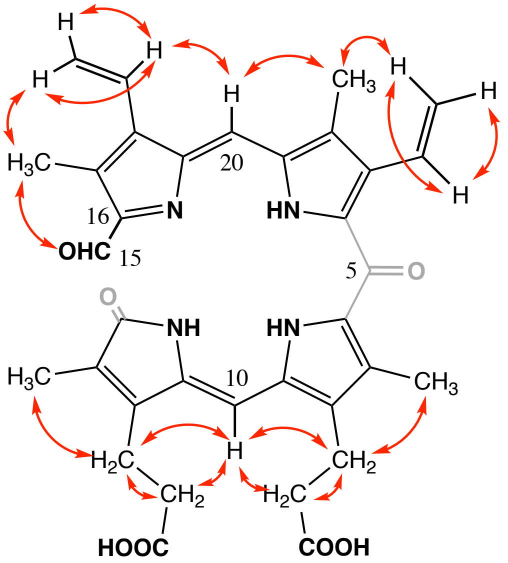

Figure S5. NMR spectra of 16-formyl-δ-staphylobilin (product Y). (C) NOESY spectrum, and (D) observed NOESY correlations.

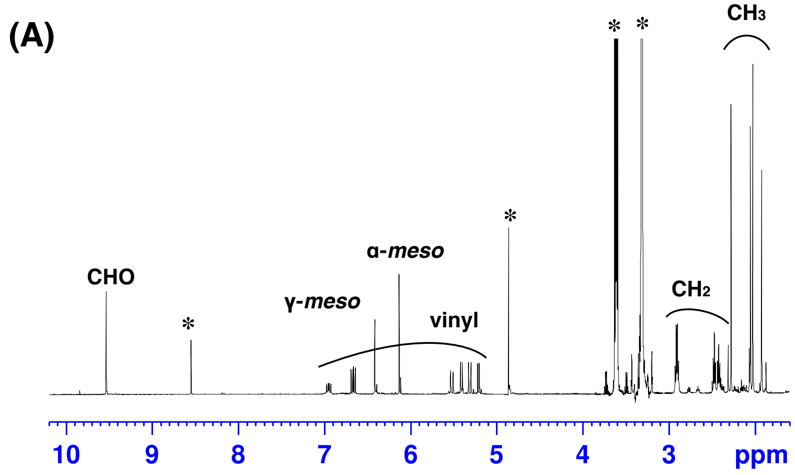

^{1}H-NMR (600 MHz, CD_3OD): δ = 9.56 (s, 1H, 4-CHO), 6.97 (dd, J = 17.7, 11.5 Hz, 1H, 3-vinyl-CH), 6.69 (dd, J = 17.8, 11.6 Hz, 1H, 18-vinyl-CH), 6.44 (s, 1H, 10C-H), 6.16 (s, 1H, 20C-H), 5.54 (dd, J = 17.8, 1.6 Hz, 1H, 3-vinyl-CH_2, *trans*), 5.43 (dd, J = 11.5, 1.7 Hz, 1H, 3-vinyl-CH_2, *cis*), 5.34 (dd, J = 17.8, 1.7 Hz, 1H, 18-vinyl-CH_2, *trans*), 5.24 (dd, J = 11.6 and 1.7 Hz, 1H, 18-vinyl-CH_2, *cis*), 2.94 and 2.49 (m, 4H, 8- or 12-CH_2), 2.92 and 2.44 (m, 4H, 12- or 8-CH_2), 2.30 (s, 3H, 17-CH_3), 2.08 and 1.94 (s, 6H, 7- and 13-CH_3), 2.05 (s, 3H, 2-CH_3)

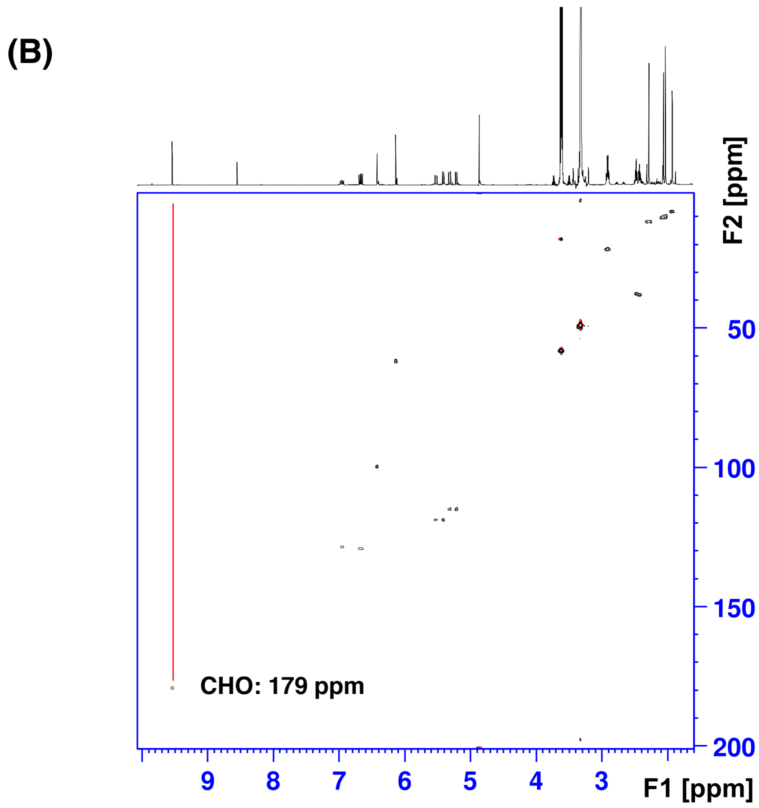

Figure S6. NMR spectra of 4-formyl-β-staphylobilin (product Z). (A) ^{1}H-NMR and (B) HSQC spectra (*continued*).

(C)

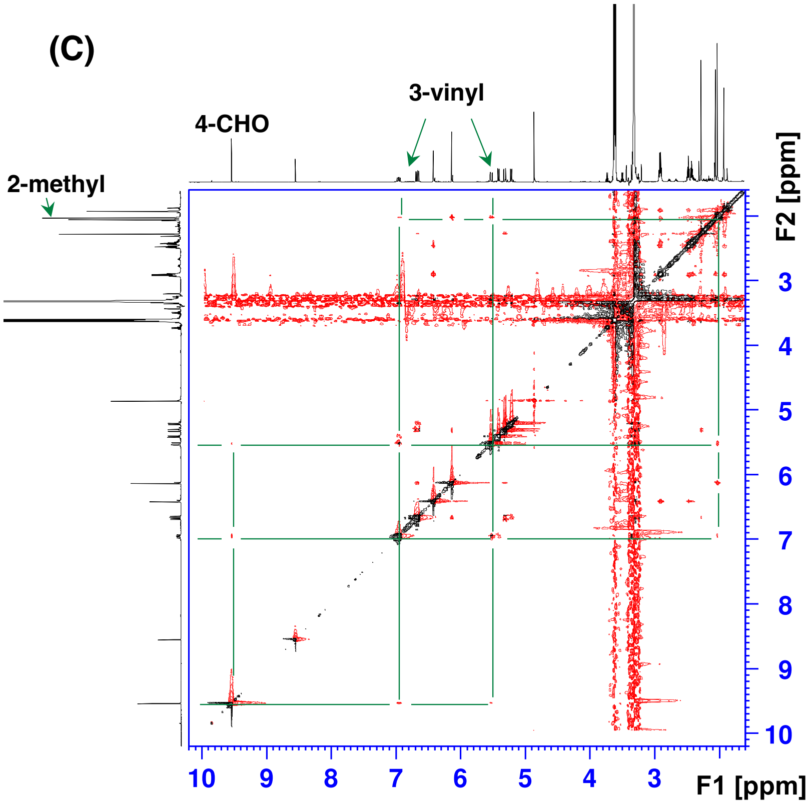

(D)

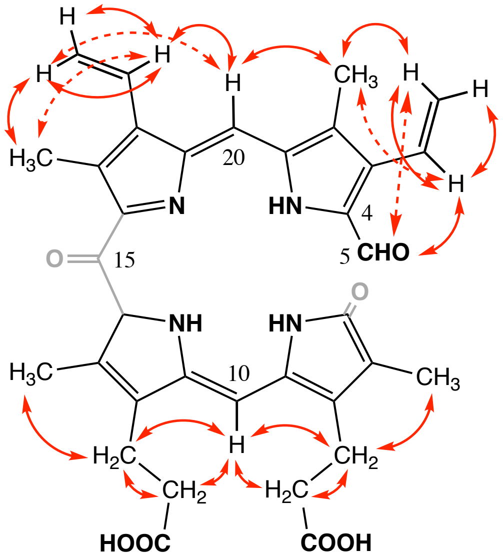

Figure S6. NMR spectra of 4-formyl-β-staphylobilin (product Z). (C) NOESY spectrum, and (D) observed NOESY correlations.

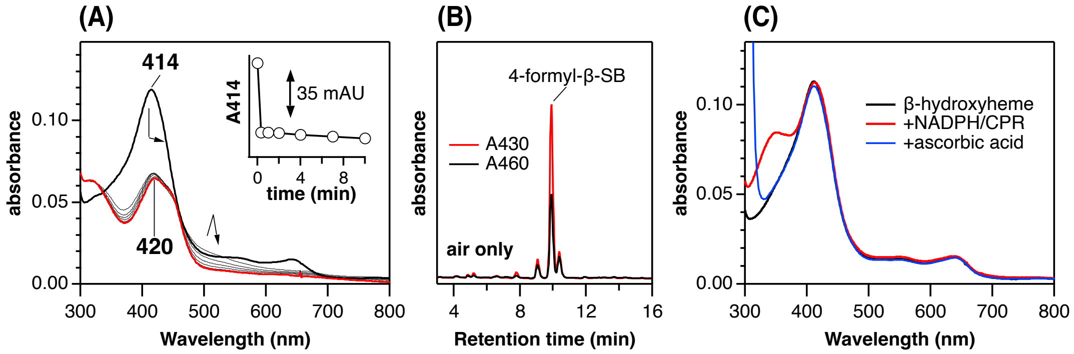

Figure S7. Reactions of hydroxyheme complexes of IsdG in 0.1 M potassium phosphate, pH 7.5 at 30 °C. (A) Absorption spectral change in reaction of δ-hydroxyheme-IsdG with O_2. (*inset*) Absorbance change at 414 nm. (B) HPLC chromatogram of air-oxidized δ-hydroxyheme-IsdG without reduction. (C) Absorption spectra of β-hydroxyheme-IsdG after 5 min of incubation with 0.5 μM CPR or 5 mM L-ascorbic acid in the absence of O_2.

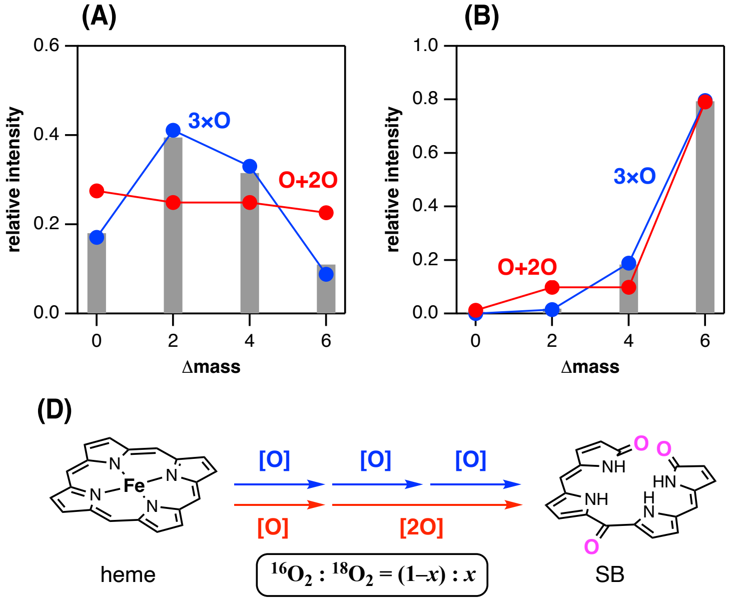

^{18}O	Δmass	3×O	O+2O
0	0	$(1-x)^3$	$(1-x)^2$
+1	2	$3x(1-x)^2$	$x(1-x)$
+2	4	$3x^2(1-x)$	$x(1-x)$
+3	6	x^3	x^2

Figure S8. Discrimination of two possible oxygenation modes for SB formation by IsdG. (A–C) Mass increase of staphylobilin (SB) in labeling with different mixing ratios of $^{16}O_2/^{18}O_2$ (*gray bars*) and their best fits with two reaction models (*blue* and *red lines*). (D) Two possible reaction models for inserting three oxygen atoms. In "O×3" mode (*blue*), one oxygen atom is added three times. In the "O+2O" mode, one and two oxygen atoms are inserted in two O_2 reactions, regardless of their order. The table summarizes the theoretical mass distribution in each model when the fraction of $^{18}O_2$ is x. These equations were used for the fittings in (A)–(C). The samples were prepared by single turnover degradation of 6 μM heme-IsdG with 1.25 mM ascorbic acid in the anaerobic glove box.

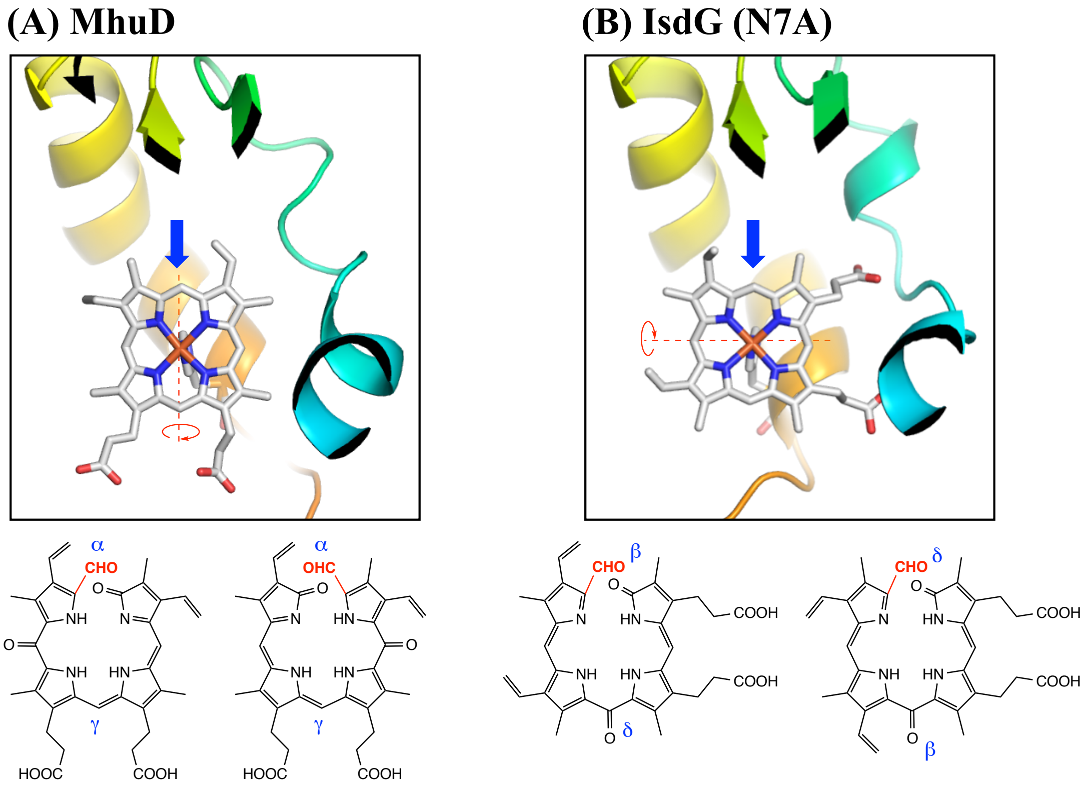

Figure S9. Heme pocket structures and tetrapyrrole products of MhuD (A) and IsdG (B). Crystal structures of ferric heme complexes of MhuD (cyanide-bound form, PDB: 4NL5) and N7A IsdG (PDB: 2ZDO). Possible dioxygenation sites and pseudo-two-fold axes of heme are indicated by *blue arrows* and *red dotted lines*, respectively.
